# Research on the social integration and depression risk of middle-aged and older adults with multiple chronic conditions in China

**DOI:** 10.3389/fpubh.2025.1559090

**Published:** 2025-04-04

**Authors:** Weijian Deng, Simin Yang, Xin Ouyang, Tao Jiang, Junmin Zhu, Feng Yang

**Affiliations:** ^1^School of Public Health, Guilin Medical University, Guilin, China; ^2^School of Humanities and Management, Guilin Medical University, Guilin, China

**Keywords:** multiple chronic conditions (MCCs), social integration, economic integration, relational integration, community integration, depression risk, middle-aged and older adults

## Abstract

**Objective:**

The aging of the global population is intensifying, and the issue of depression among middle-aged and older individuals in China has garnered significant attention. Social integration is considered closely related to the risk of depression in patients with multiple chronic conditions (MCCs). This study aims to explore the relationship between different dimensions of social integration and depression in middle-aged and older individuals.

**Methods:**

This study utilized data from the 2018 China Health and Retirement Longitudinal Study (CHARLS), including 2,901 middle-aged and older individuals with MCCs. Depression risk was assessed using a depression scale. Social integration was measured across three dimensions: economic integration, relational integration, and community integration. Statistical analyses were conducted using Logit and Tobit models to explore the relationship between social integration and depression among middle-aged and older individuals.

**Results:**

There was no significant association between overall social integration and the risk of depression, but economic integration and community integration were negatively correlated with the risk of depression, and relational integration was positively correlated with the risk of depression, and this relationship was heterogeneous among different demographic characteristics. Sensitivity analysis and subgroup analysis further confirmed the robustness of the results.

**Conclusion:**

Mental health interventions for middle-aged and older adults MCCs patients should consider the multi-dimensional characteristics of social integration. It is recommended to improve the economic status of the middle-aged and older adults, improve the quality of family and social interactions, and pay attention to the construction of the community environment. And further explore the interaction of each dimension and its applicability in different contexts.

## 1 Introduction

The continuous growth of the global population, coupled with declining fertility rates and increased life expectancy, has led to unprecedented aging worldwide, with significant variations in future demographic trends across different regions and countries (1). In China, by the end of 2023, the older adults population aged 60 and above reached 296.97 million, accounting for 21.1% of the total population; those aged 65 and above constituted 15.4%, with a population dependency ratio of 22.5%. It is projected that China will enter a stage of severe aging around 2035, with the scale and proportion of the older adults population peaking around 2050 (2). Against this backdrop, health issues within the middle-aged and older adults cohorts are emerging, with depression as a prototypical mental health disorder, representing a significant public health challenge for China (3, 4). Among the older adults, depression predominantly affects individuals with chronic diseases and cognitive impairments. When aging converges with psychosocial adversities, it tends to heighten susceptibility to depression among middle-aged and older individuals—a factor often overlooked (5, 6). Consequently, screening for mental health issues in this demographic, alongside identifying specific influencing factors, is of paramount importance.

Physiological decline, unhealthy lifestyles, and psychosocial factors contribute to a high prevalence of chronic diseases, such as hypertension and diabetes, among the middle-aged and older adults population. Furthermore, patients with chronic diseases are frequently accompanied by two or more comorbidities, known as multiple chronic conditions (MCCs) (7). With each additional chronic condition, older adults with MCCs have a 50% increased risk of further functional decline and accumulate a disproportionate health and cost burden (8, 9). Numerous studies demonstrate a positive correlation between the number of chronic diseases and the incidence of depression (10–13). This association is likely due to the significant impact of MCCs on quality of life, stemming from the need for regular medication, frequent check-ups, and a higher degree of functional dependence. The more pronounced decline in physical function often leads to increased anxiety and worry about their health status, fostering negative emotions and consequently triggering or exacerbating depressive symptoms.

Social integration, broadly defined in sociology as the opposite of social isolation—the detachment from social relationships, institutional connections, or community participation—emerged to explain immigrant behavior, adaptation, acculturation, and self-identity (14, 15). It has since been more widely applied to describe the process by which individuals establish meaningful connections within their social and cultural environments. Given that social integration is a multidimensional concept, this study adopts the framework proposed by Amu Mose et al. to systematically explore social integration in older adults across a broader spectrum (16). This framework comprehensively encompasses the core dimensions of social integration and provides appropriate measurement methods. Within this framework, social integration is categorized into three key dimensions. The first dimension, economic integration, refers to an individual’s level of participation in economic activities, which is reflected through their living environment and consumption patterns. This dimension highlights an individual’s position and capacity within the socio-economic structure and is essential for maintaining a stable and secure life. The second dimension, relational integration, focuses on the extent to which individuals are embedded within their social relationships, emphasizing their connections with social networks such as family and friends. This includes the receipt of emotional support and the frequency of interpersonal interactions. The third dimension, community integration, underscores active participation in community activities, such as engagement in interest groups, volunteer work, or other forms of civic involvement. It reflects individuals’ sense of belonging and integration within the broader social environment. Together, these dimensions provide a comprehensive framework for understanding the multifaceted nature of social integration, particularly in its application to specific populations such as older adults.

Measurement of social integration necessitates a focus on both individual capabilities and societal opportunities, particularly crucial for vulnerable populations experiencing poverty and poor health (17). Existing evidence strongly suggests a close association between social integration and depression risk among the middle-aged and older adults. Specifically, greater social integration is linked to lower depression risk. While some studies focus on family and friend interactions (18–21), most research operationalizes social integration through participation in social activities and interest groups, as well as adaptation to mainstream cultural values and norms (22–24). These studies largely concur on the positive impact of social integration on mental well-being. However, current research on the effect of social integration on depression risk in the middle-aged and older adults primarily focuses on limited, single concepts and dimensions, such as social participation, social networks, and social support, neglecting the multifaceted nature of social integration. Furthermore, multidimensional indicators in some studies may not be adequately tailored to the specific needs of older adults, overlooking individual characteristics. Few studies have specifically assessed the impact of different dimensions of social integration on depression risk among older adults, particularly those with MCCs.

This study aims to assess the level of social integration, its three dimensions, and their correlation with depression risk among middle-aged and older adults with MCCs. The study will also examine the specific effects of social integration across different demographic characteristics. Integrating social integration strategies into interventions for middle-aged and older adults with MCCs will contribute theoretically to improving mental health, enhancing quality of life, promoting overall well-being, and advancing healthy aging in this population.

## 2 Methods

### 2.1 Data sources

**Figure 1:**
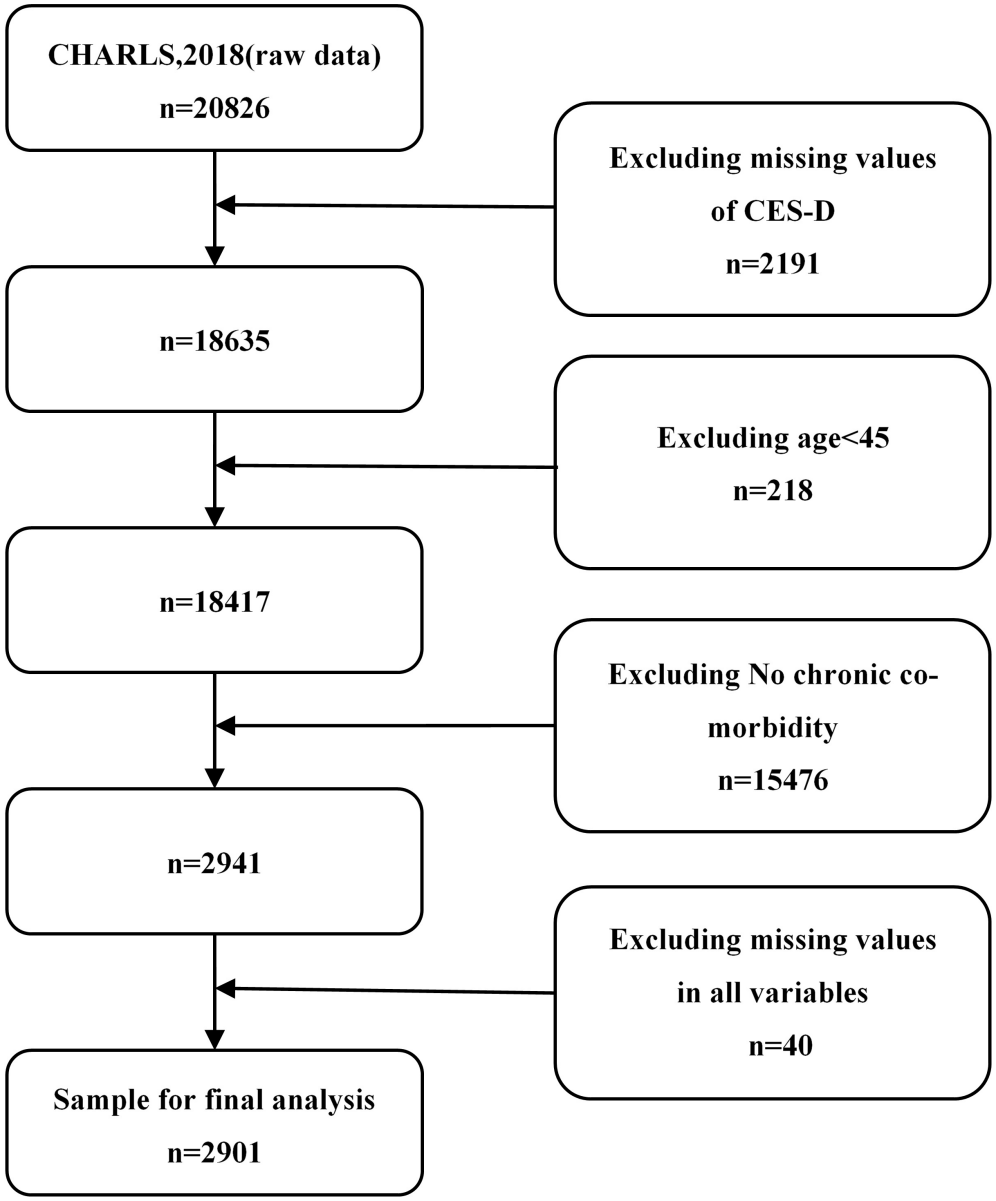
Flowchart of the selection process of the research object.

The China Health and Retirement Longitudinal Study (CHARLS) is a nationally representative longitudinal survey designed to collect high-quality micro-level data on families and individuals aged 45 and above in China. The study aims to analyze the implications of China’s aging population, including assessments of the social, economic, and health status of community residents. The national baseline survey was conducted in 2011, with subsequent follow-up surveys conducted continuously. This project received ethical approval from the Peking University Biomedical Ethics Committee, and informed consent was obtained from all participants prior to questionnaire completion. The CHARLS dataset is available for download online at https://charls.charlsdata.com/pages/data/111/zh-cn.html. This study utilized data from CHARLS 2018. One of the inclusion criteria was that participants had to be diagnosed with multiple chronic conditions (MCCs). The CHARLS survey question, “Have you been diagnosed with this chronic disease by a doctor?,” was used to determine the presence of chronic conditions. Fourteen chronic conditions were considered: hypertension, dyslipidemia (hyperlipidemia or hypolipidemia), diabetes or hyperglycemia, cancer (malignant neoplasms), chronic lung disease, liver disease, heart disease, stroke, kidney disease, stomach or digestive system disease, mood and mental health problems, memory-related diseases, arthritis or rheumatism, and asthma. Individuals reporting two or more of these conditions were classified as having MCCs. After variable selection and data cleaning, a final sample of 2,901 participants was included in the analysis. The detailed process is shown in Figure 1.

### 2.2 Variables

#### 2.2.1 Dependent variable

Depression risk was measured using the 10-item Center for Epidemiological Studies Depression Scale (CES-D-10) from CHARLS 2018, which was developed by Radloff. The Cronbach’s alpha coefficient of the CES-D-10 was 0.816 in our study sample, which had a high internal reliability to consistently measure depressive symptoms in this study. A score was assigned based on responses to ten questions assessing negative emotions and behaviors. Items such as “I was bothered by things that do not usually bother me,” “I had trouble keeping my mind on what I was doing,” “I felt depressed,” “I felt everything I did was an effort,” “I felt fearful,” “My sleep was restless,” “I felt lonely,” and “I could not get going” were scored 1, 2, 3, and 4 for “rarely or never (<1 day),” “some or a little of time (1–2 days),” “occasionally or a moderate amount of the time (3–4 days),” and “most or all of the time (5–7 days),” respectively. The items “I was happy” and “I felt hopeful about the future” were reverse-scored. The CES-D total score ranged from 0 to 30. Scores <10 were assigned a value of 0 (no depression risk), while scores ≥10 were assigned a value of 1 (depression risk).

#### 2.2.2 Independent variables

Social integration comprised three dimensions: economic integration, relational integration, and community integration, based on the definitions provided by Amu Mose et al. (16). Economic integration included housing structure and annual per capita household consumption level. A composite score representing low, medium, and high levels (0, 0.5, and 1, respectively) was created using the average of these two variables. Relationship integration is measured by the frequency of interactions between middle-aged and older adults with their non-cohabiting children (meeting/telephone calls/text messages/WeChat/letters/e-mails) and interactions with friends. For family interaction, responses to the question regarding frequency were coded as follows: “almost never” (0), “every half-month or less” (1), “1–3 times per week” (2), and “almost every day” (3). Missing values were coded as 0. For friend interaction, the question “How often have you interacted with friends in the past month?” was used, with the following coding: “not often” (1), “almost every week” (2), “almost daily” (3). Missing values were coded as 0 (“no participation”). The average of the child interaction score and friend interaction score was used as the relational integration score (range 0–3). A higher score indicates a greater degree of relational integration. Community integration was measured using three items: “How often have you played mahjong, chess, cards, or went to community club in the past month?,” “How often have you danced, exercised, or practiced Qigong in the past month?,” and “How often have you participated in community organization activities in the past month?.” Each item was scored using the same 0, 1, 2, 3 scale as above. The total social integration score was the sum of the three dimensions (range 0–7), with higher scores reflecting greater social integration.

#### 2.2.3 Control variables

To account for individual characteristics, age, gender, residence, marital status, and education level were included as control variables. Age was categorized as “<60 years” and “≥60 years”; marital status as “married” and “unmarried” (including divorced, widowed, single, or cohabiting); education level as “illiterate,” “primary school graduate,” and “junior high school and above”; and residence as “urban” and “rural.”

### 2.3 Statistical analysis

Descriptive statistics, including frequencies and percentages for categorical variables and means and standard deviations for continuous variables, were used to characterize the participants’ baseline characteristics. To analyze the relationship between social integration and its three dimensions and the risk of depression (y = 0/y = 1) among Chinese middle-aged and older adults, logistic regression models were employed. Given the potential non-linear relationship, we explored this possibility within the models. To assess the impact of social integration on the severity of depression, a Tobit regression model was utilized. This addressed the censored nature of the continuous CES-D scores, treating scores below 10 as censored at 0. To investigate potential heterogeneity in the association between social integration and depression risk across different demographic characteristics, interaction terms were included in the models for gender, age, residence, marital status, and education level with each dimension of social integration (economic, relational, and community). Robustness checks were performed to assess the stability of the results under different model specifications. Specifically, sensitivity analyses included: (1) ordered logistic regression, accounting for the ordinal nature of the depression outcome, with multivariate adjustment; and (2) a replication of the main analysis excluding participants with hypertension, given its high prevalence and established association with depression (25), to examine the relationship between other comorbid conditions and depression. All statistical analyses were conducted using Stata 17.0.

## 3 Results

### 3.1 Participant characteristics

Of the 2,901 middle-aged and older adults with MCCs included in this study, over half (58.81%) were aged 60 years or older, indicating a predominance of older adults compared to middle-aged adults according to Chinese age classifications. The sample was roughly evenly divided between males and females. A significantly larger proportion of participants resided in rural areas (66.49%) compared to urban areas (33.51%). The majority of participants were married (83.52%), while 16.48% were divorced, widowed, or in other marital statuses. A substantial proportion (41.19%) of participants were illiterate, with 22.54% having completed primary school and 36.26% having completed junior high school or higher. The mean total social integration score was 1.52 ± 0.93. The majority (63.08%) of participants exhibited a moderate level of economic integration. Among the participants with MCCs, the prevalence of depressive symptoms showed considerable variability, with 1,270 individuals (43.78%) exhibiting depressive symptoms (see Table 1).

**Table 1:** Variable definitions and basic characteristics.

Variables	Definitions	*N*(%)/Mean ± SD
Depression	Measured by cesd scale in CHARLS 2018, the score ranges from 0 to 30 and depression values increase with higher scores.	9.30 ± 7.48
	<10 scores = 0	1,631 (56.22)
	≥10 scores = 1	1,270 (43.78)
Economic integration	Housing structure + annual consumption expenditure per capita	
	Low = 0	306 (10.55)
	Medium = 0.5	1,830 (63.08)
	High = 1	765 (26.37)
Relational integration	Family Interaction + Friends Interaction	0.77 ± 0.71
Community integration		0.17 ± 0.37
Social integration	Economic integration + relational integration + community integration	1.52 ± 0.93
Age	<60 years = 1	1,111 (38.30)
	≥60 years = 2	1,790 (61.70)
Gender	Male = 1	1,397 (48.16)
	Female = 0	1,504 (51.84)
Residence	Urban = 1	972 (33.51)
	Rural = 2	1,929 (66.49)
Marital status	Married = 1	2,423 (83.52)
	Non-married = 0	478 (16.48)
Education	Illiteracy = 1	1,195 (41.19)
	Primary school = 2	654 (22.54)
	Junior high school and above = 3	1,052 (36.26)

### 3.2 Binary logistic regression results

The results from Models 1 and 2 indicate no significant association between overall social integration and individual depression risk. However, when examining the three dimensions of social integration separately in Model 3, we find that economic integration and community integration are significantly negatively associated with depression risk (*p*<0.05). This suggests that higher levels of integration in these two dimensions correspond to lower depression risk. Conversely, relational integration shows a positive correlation with depression risk (OR = 0.218, 95%CI: 0.110–0.325, *p* < 0.05), suggesting that increased relational integration may heighten the risk of depressive symptoms. In Model 4, even after controlling for all covariates, the negative associations between economic and community integration and depression risk remain robust (OR = 0.194, 95%CI: 0.083–0.305, *p* < 0.05). However, the impact of relational integration appears particularly pronounced, indicating that this dimension may play a critical role in the development of individual depression and is strongly linked to depressive symptoms. Among sociodemographic variables, being female, under the age of 60, residing in rural areas, and being illiterate are identified as risk factors for depressive symptoms. The relationship between marital status and depression risk is not significant (see Tables 2, 3).

**Table 2:** Logistic regression analysis of the relationship between total social integration and risk of depression in middle-aged and older adults.

Variable	Model 1		Model 2	
	OR(95%)	*P*	OR(95%)	*P*
Social integration	−0.033(−0.112,0.046)	0.416	0.024(−0.059,0.107)	0.576
Gender(ref: Female)				
Male			−0.444(−0.602,−0.285)	<0.001
Age(ref: 45~59 years)				
≥60 years			−0.216(−0.378,−0.055)	0.009
Residence(ref: Urban)				
Rural			0.570(0.398,0.741)	<0.001
Marital status(ref: Non-married)				
Married			0.009(−0.201,0.219)	0.935
Education(ref: Illiteracy)				
Primary school			−0.144(−0.345,0.057)	0.161
Junior high school and above			−0.456(−0.649, −0.262)	<0.001

*p < 0.1, **p < 0.05, ***p < 0.01.

**Table 3:** Logistic regression analysis of the relationship between three dimension of social integration and risk of depression in middle-aged and older adults.

Variable	Model 3		Model 4	
	OR(95%)	*P*	OR(95%)	*P*
Economic integration(ref: Low)				
Medium	−0.332(−0.576,−0.088)	0.008	−0.240(−0.490,0.010)	0.060
High	−0.655(−0.926,−0.384)	<0.001	−0.338(−0.628,−0.048)	0.022
Relational integration	0.218(0.110,0.325)	<0.001	0.194(0.083,0.305)	0.001
Community integration	−0.426(−0.639,−0.212)	<0.001	−0.318(−0.537,−0.100)	0.004
Gender(ref: Female)				
Male			−0.448(−0.607,−0.289)	<0.001
Age(ref: 45~59 years)				
≥60 years			−0.221(−0.384,−0.059)	0.008
Residence(ref: Urban)				
Rural			0.478(0.299,0.658)	<0.001
Marital status(ref: Non-married)				
Married			0.082(−0.132,0.295)	0.453
Education(ref: Illiteracy)				
Primary school			−0.125(−0.327,0.077)	0.224
Junior high school and above			−0.415(−0.610,−0.219)	<0.001

*p < 0.1, **p < 0.05, ***p < 0.01.

### 3.3 Tobit regression results

The preliminary analysis results from Models 7 and 8 indicate no significant association between overall social integration and depression levels. However, Models 9 and 10 reveal significant correlations between all three dimensions of integration and depression severity (*p* < 0.05). Specifically, enhanced economic integration and deeper community integration were found to significantly reduce the likelihood of severe depressive symptoms among middle-aged and older individuals. Conversely, strengthened relational integration was associated with an increased risk of depression. Furthermore, our findings suggest that participants who were over 60 years old, resided in urban areas, and possessed higher educational levels were likely to experience lower levels of depressive symptoms (see Table 4).

**Table 4:** Tobit regression analysis of the relationship between three dimension of social integration and risk of depression in middle-aged and older adults.

Variable	Model 7 (unadjusted)	Model 8	Model 9 (unadjusted)	Model 10
	OR	OR	OR	OR
Economic integration(ref: Low)				
Medium			−3.465***	−2.419**
High			−7.051***	−3.548***
Relational integration			2.039***	1.643***
Community integration			−5.093***	−3.854***
Social integration	−0.648	−0.106		
Gender(ref: Female)				
Male		−4.560***		−4.584***
Age(ref: 45~59 years)				
≥60 years		−1.769***		−1.839**
Residence(ref: Urban)				
Rural		5.453***		4.480***
Marital status(ref: Non-married)				
Married		−0.598		0.085
Education(ref: Illiteracy)				
Primary school		−1.918**		−1.697*
Junior high school and above		−5.025***		−4.531***

*p < 0.1, **p < 0.05, ***p < 0.01.

### 3.4 Sensitivity and subgroup analyses

#### 3.4.1 Sensitivity analysis

We conducted a sensitivity analysis by reclassifying the original binary outcome variable into three categories: mild depression, severe depression, and no depression. We then performed an ordinal logistic regression. Additionally, we excluded participants with hypertension (*n* = 515) and executed the original regression model. In both sensitivity analyses, after adjusting for age, gender, residence, marital status, and education level, the results remained consistent with the primary analysis. Specifically, the significant associations between the three dimensions of social integration and depression risk persisted. Economic integration and community integration were inversely associated with depression risk, whereas relational integration was positively associated. These associations exhibited significant differences upon variable adjustment (see Table 5).

**Table 5:** Sensitivity analysis results.

	Variable	OR(95%)	*P*	OR(95%)	*P*
Model 11^a^	Economic integration(ref: Low)				
	Medium			−0.251(−0.486,−0.017)	0.036
	High			−0.347 (−0.621,−0.073)	0.013
	Relational integration			0.149(0.045,0.254)	0.005
	Community integration			−0.367(−0.575,−0.158)	0.001
	Social integration	−0.017(−0.096,0.062)	0.676		
Model 12^b^	Economic integration(ref: Low)				
	Medium			−0.213(−0.491,0.066)	0.134
	High			−0.279(−0.600,0.042)	0.088
	Relational integration			0.187(0.064,0.310)	0.003
	Community integration			−0.388(−0.626,−0.150)	0.001
	Social integration	0.006(0.085,0.097)	0.896		

a Ordered logistic regression based on multivariate models. Models adjusted for gender, age, residence, marital status, and education level.

b Logistic regression based on a multivariate model after removal of hypertensive middle-aged and older adults patients (*n* = 2,386). Models adjusted for gender, age, residence, marital status, and education level.

#### 3.4.2 Subgroup analysis

Building on the logistic and Tobit regression outcomes, we further explored the association between social integration across its three dimensions and depression risk among target populations with varying characteristics. For age, the associations of economic integration and community integration with depression were more pronounced in males over 60 years old, with high levels of economic integration exerting a more substantial impact on depressive symptoms (OR = −0.632, *p* < 0.05). No significant associations were found among younger and female participants. Regarding residence, economic integration was significantly related to depression risk in middle-aged and older individuals residing in rural areas, but not in urban residents. Both rural and urban community integration reduced the participants’ depression risk. Among the three dimensions, the effects of relational integration contrasted with those of economic and community integration, showing a significant positive association with depression risk across age and gender. Rural middle-aged and older individuals were more likely to experience depressive symptoms due to relational integration than their urban counterparts (OR = 0.211, *p* < 0.05). In terms of education level, all three dimensions of integration were associated with depression risk among illiterate participants. High levels of economic integration potentially reduced depression risk for individuals with elementary education (OR = −0.601, *p* < 0.1), while community integration had a significant impact in the higher education group (OR = −0.295, p < 0.1). Overall, the influence of social integration on the mental health of middle-aged and older individuals exhibited heterogeneity across different demographic groups. The results of the differences between the groups are shown in Tables 6–9.

**Table 6:** Association between social integration and risk of depression, stratified by age.

Variable	45–59 years(*n* = 1,111)		≥60 years(*n* = 1790)	
	Coefficient	S.E.	Coefficient	S.E.
Economic integration(ref: Low)				
Medium	−0.066	0.224	−0.312**	0.156
High	0.076	0.246	−0.632***	0.190
Relational integration	0.269***	0.095	0.171**	0.072
Community integration	−0.103	0.172	−0.503***	0.150
Cons	−0.031	0.336	−0.027	0.213

*p < 0.1, **p < 0.05, ***p < 0.01. Adjusted for gender, residence, marital status and level of education.

**Table 7:** Association between social integration and risk of depression, stratified by gender.

Variable	Male(*n* = 1,397)		Female(*n* = 1,504)	
	Coefficient	S.E.	Coefficient	S.E.
Economic integration(ref: Low)				
Medium	−0.225	0.182	−0.254	0.181
High	−0.427**	0.213	−0.261	0.208
Relational integration	0.230***	0.087	0.187**	0.076
Community integration	−0.569***	0.186	−0.173	0.142
Cons	0.103	0.291	−0.286	0.262

*p < 0.1, **p < 0.05, ***p < 0.01. Adjusted for age, residence, marital status and level of education.

**Table 8:** Association between social integration and risk of depression, stratified by residence.

Variable	Urban(*n* = 972)		Rural(*n* = 1929)	
	Coefficient	S.E.	Coefficient	S.E.
Economic integration(ref: Low)				
Medium	−0.209	0.400	−0.228*	0.136
High	−0.182	0.403	−0.470***	0.173
Relational integration	0.142	0.109	0.211***	0.067
Community integration	−0.329*	0.169	−0.316**	0.150
Cons	−0.251	0.453	0.595	0.199

*p < 0.1, **p < 0.05, ***p < 0.01. Adjusted for gender, age, marital status and level of education.

**Table 9:** Association between social integration and risk of depression, stratified by education.

Variable	Illiteracy(*n* = 1915)		Primary school(*n* = 654)		Junior high school and above(*n* = 1,052)	
	Coefficient	S.E.	Coefficient	S.E.	Coefficient	S.E.
Economic integration(ref: Low)						
Medium	−0.201	0.172	−0.411	0.274	−0.094	0.282
High	−0.587***	0.223	−0.601*	0.312	0.033	0.295
Relational integration	0.271***	0.087	0.151	0.124	0.136	0.096
Community integration	−0.410**	0.196	−0.268	0.230	−0.295*	0.169
Cons	−0.012	0.287	0.252	0.404	−0.562	0.359

*p < 0.1, **p < 0.05, ***p < 0.01. Adjusted for gender, age, residence and marital status.

## 4 Discussion

This study focuses on middle-aged and older individuals with multiple chronic conditions (MCCs), aiming to elucidate the potential relationship between social integration and depression. Our findings underscore the significant associations between the three dimensions of social integration—economic integration, relational integration, and community integration—and the risk of depression among MCCs patients in this demographic. Specifically, economic and community integration have a positive impact in reducing depression risk. However, relational integration unexpectedly correlates positively with the severity of depression, which contrasts with previous studies suggesting that family and friend support benefits middle-aged and older individuals in combating depression (26). This discrepancy suggests that different types of social integration may play distinct roles in the mental health of MCCs patients. Given this, we focus on exploring the mechanisms through which each dimension of social integration influences depression in middle-aged and older individuals.

### 4.1 The impact of economic integration on depression

Many studies have established the link between housing environment and mental health, showing that depressive symptoms are significantly associated with various aspects of the housing conditions, including material conditions, social relationships, and personal psychological experiences (27, 28). The physical structure of housing, as one of its environmental attributes, works in conjunction with the social and psychological aspects to affect the mental health of individuals (29). Two studies of older people in rural China have shown that unimproved housing materials other than concrete and steel/brick and wood create poor physical conditions for the emergence of depression in older people, and that housing conditions such as polluting cooking fuels, latrines with no seating capacity, and the absence of bathing facilities are also significantly associated with depression, with gender differences (30, 31). Differences in housing structure can lead to variations in living arrangements and housing density, affecting the level of social support and its impact on depression risk and severity among middle-aged and older individuals (32, 33). Furthermore, annual per capita consumption expenditure reflects the socioeconomic status of this demographic, where favorable living conditions and expenditures on daily needs and recreation can enhance mental health. For middle-aged and older individuals, especially those with illnesses, healthcare-related expenditures like medical insurance constitute a significant portion of spending. The quality of health insurance and healthcare services is crucial in alleviating depression among those with chronic illnesses (34). Economically disadvantaged middle-aged and older individuals lacking pension and insurance plans may face catastrophic health expenditures, leading to debt and poverty, which in turn elevate the risk of depression (35).

In this study, the reduction in depression risk attributed to economic integration, as measured by housing structure and annual per capita consumption expenditure, is predominantly observed in older age groups. This pattern is likely due to increased vulnerability, declining physiological and psychological adaptability, and economic uncertainties post-retirement, making those nearing retirement age more susceptible to depression, self-harm, and suicide induced by economic recession (36). Research indicates that women are more frequently represented in adverse socioeconomic health conditions than men, such as employment status, family poverty, and educational attainment (37). As a result, women may bear more roles in family and society, potentially diminishing the positive effects of economic integration on their mental health. In contrast, men may rely more heavily on socioeconomic status to gain social support (38). Additionally, depression among middle-aged and older adults men may stem from decreased social support and heightened need for connection following the death of a spouse (39). For middle-aged and older individuals living in rural areas with lower educational levels, poorer economic integration increases depression risk. This reflects inequalities in mental health and resource allocation between rural and urban areas (40), as rural regions often have inferior housing conditions and lower living standards. Lower educational levels may imply limited health literacy and coping skills, increasing susceptibility to medical conditions and psychological disorders (41). These factors contribute to diminished social support, thereby raising depression risk.

### 4.2 The impact of community integration on depression

In the context of global mental health, active participation in community and civic life is recommended as a key concept for measuring social integration worldwide (17). This study focuses on social integration in terms of middle-aged and older individuals’ participation in community collective activities, rather than interactions with family and friends. These activities include indoor recreational events, outdoor exercises, and organized group activities. Community resources provide effective services and foster positive interactions, offering opportunities for physical and mental exercises, which are protective factors against depression (42). Literature indicates that social participation among older adults women in China is lower than that of older adults men, with this gap widening with age (43). Our findings corroborate this, showing that community integration significantly combats depression only among older adults men (≥60 years). This may be because retired men have more time and opportunities to engage in neighborhood and community interactions, while women might participate less due to more household responsibilities and internal social activities (44). The positive relationship between participation in social groups, hobby groups, and community organizations and depressive symptoms is more pronounced and flexible for older adults men than women (45). The experience of positive emotions from social participation has a greater impact on men than women (46). Furthermore, the effect of community integration does not vary by residence (rural or urban), suggesting that while the forms of community integration may differ, older individuals universally benefit from community activities, reducing depression risk. Educational attainment may also influence the psychological benefits individuals derive from community activities. Variations in education levels shape diverse cultural and value systems, leading to differences in their engagement with community activities (47). Community integration can alleviate depression among illiterate individuals, potentially because those living alone in rural areas with low educational levels have limited access to community resources and lack alternative means of interaction, making them more reliant on community activities to meet social needs. Community stability is crucial for social integration; as age and community stability increase, so does social integration. Therefore, creating a safe high-quality community environment is essential for promoting social integration and the mental health of the older adults (48).

### 4.3 The impact of relational integration on depression

Unlike previous studies, our research finds a positive correlation between relational integration and the risk of depression among middle-aged and older individuals. This relationship does not vary by age or gender, but when considering residence and educational level, it is particularly significant among those living in rural areas and those who are illiterate. This finding prompts us to reconsider: frequent interactions do not necessarily equate to high-quality interactions. Middle-aged and older individuals may experience negative social support, facing more adverse family and social interactions.

Beyond the adverse psychological states caused by illness, the transition of social roles among middle-aged and older individuals is a significant practical cause for exacerbating depression. In the context of China’s aging population, changes in family structure have weakened the familial support functions for empty-nest older adults living alone, thereby increasing the demand for social support (49). Specifically, intimate partnerships may increase the susceptibility of the older adults to depression due to the severe consequences of losing companionship, which in turn reduces future interpersonal relationships. Conversely, active community participation can enhance social connections among the older adults (50). Consequently, in rural areas predominantly inhabited by left-behind and empty-nest older adults, middle-aged and older individuals rely more on stable interactions within the community. Furthermore, the theory of relational burden and stress provides an explanation for the causes of depressive emotions in middle-aged and older individuals—friends and family may become sources of relational burden by making excessive demands (51). In communities where these relational burdens are concentrated, the protective role of collective efficacy is particularly significant (52). This positive influence of collective efficacy on mental health is especially evident among the older adults, who are more inclined to increase their participation and time investment in their residential communities during later life (53). A study on the mental health of those living alone supports this phenomenon: if the negative impacts on mental health caused by social integration are not offset or compensated by the positive aspects of social relationships, such integration may instead lead to psychological distress (54). According to this theory, if positive community integration can compensate for negative relational integration, depression among middle-aged and older individuals with MCCs could be alleviated.

Given that respondents are patients with chronic comorbidities, the negative impact of low-quality relationships on mental health is particularly noteworthy as middle-aged and older individuals bear the heavy burden of chronic illness. A study on Hispanic cancer patients revealed that increased stressful negative interactions among family members might be proximal risk factors for the severity of depression in cancer patients, likely due to additional depressive emotions arising from illness (55). Meanwhile, negative interactions with family members may hinder patients’ help-seeking processes and limit their access to emotional and tangible support resources from family (56). Notably, even though older adults generally experience fewer negative interactions with family than younger individuals, these interactions tend to be more persistent and enduring (57). Moreover, surveys have found that despite frequent interactions, older adults receive limited support from children, and the proportion of middle-aged and older individuals expecting to rely on children’s support has decreased (58). On the other hand, the size and density of relational networks can also affect the mental health of older individuals in China. Individuals with less dense networks receive more criticism from friends and have poorer mental health (59). For illiterate rural older individuals, their passive stance in interactions with children and friends may be magnified (60), making the negative impact of relational integration on mental health more pronounced.

## 5 Limitations and strengths

The limitations of this study cannot be overlooked. The health data used in this paper are self-reported, and may be subject to respondent bias in understanding the issues, social expectation bias, etc., and causal relationships between the core variables could not be established. Although the positive correlation results suggest possible conclusions, the one-year cross-sectional data and potential biases in the measurement of social integration limit the generalizability of the findings, necessitating further data exploration and validation. Additionally, the specific impact of comorbid chronic diseases on the relationship between social integration and depression in middle-aged and older individuals remains uncertain. Nevertheless, the CHARLS is a highly representative large-scale longitudinal dataset. Based on this, we analyzed and explored the differential benefits of various forms of interaction for different population characteristics, which can serve as a foundation for investigating depression in middle-aged and older adults populations. In future research, self-reported data can be validated by combining validated data from multiple sources and other supporting tools, and it is essential to construct refined and effective models for in-depth longitudinal studies to validate and thoroughly examine the combined effects of complex comorbidity patterns and different dimensions of social integration on depression, as well as to distinguish the varying support roles of family and friends. Notably, the role of the field of artificial intelligence in promoting social integration merits attention, as it may offer new perspectives and tools for mental health management.

## 6 Conclusions and recommendations

Our research findings have emphasized the necessity to consider the multi-dimensional characteristics of social integration when designing mental health intervention measures for middle-aged and older adults patients with MCCs. Most of the current studies have shown that economic support and community support are beneficial for reducing the risk of depression and alleviating depressive symptoms among the middle-aged and older adults. However, the conclusions regarding the impact of support from family and friends on mental health are inconsistent. In our study, economic integration and community integration among the middle-aged and older adults are equally effective ways to reduce depression, while relationship integration has, to a greater extent, brought pressure and burden to them. Such negative integration may exacerbate the depressive situation. Based on these results, we put forward the following recommendations: First, governments should prioritize and expand economic assistance programs targeting low-income older adults with MCCs, including financial subsidies, pension system reforms, and the creation of age-appropriate employment opportunities, to improve their living conditions and purchasing power. Second, local governments and community organizations should invest in inclusive, age-friendly environments, such as senior activity centers, intergenerational interaction initiatives, and volunteer service programs, with a focus on ensuring accessibility for those with mobility limitations or chronic illnesses to promote active participation. Additionally, resources to support caregivers and family members, such as counseling services, caregiver support groups, and respite care programs, are urgently needed. Mental health education initiatives should be implemented to raise awareness about balancing caregiving roles with self-care, thereby improving the quality of familial and social interactions. Healthcare providers play a critical role by regularly assessing social integration levels among older adults to deliver tailored interventions. Economic and social support should also be integrated into chronic disease management, such as referring MCCs patients to financial assistance programs or community engagement activities to complement medical treatments and enhance mental health outcomes. Holistic care models that integrate mental health care, social support, and economic guidance should be adopted, with multidisciplinary teams addressing the diverse needs of older adults. Future research should explore the mechanisms through which relational integration may act as a protective factor and conduct longitudinal studies to examine the interactions between different dimensions of social integration and their long-term effects on mental health. By fostering supportive environments that enable older adults to manage chronic conditions while combating depression, we can help them achieve a more positive and fulfilling aging experience.

## Data Availability

Publicly available datasets were analyzed in this study. Data is available from the China Health and Retirement Longitudinal Study (CHARLS) database (https://charls.charlsdata.com/pages/Data/2018-charls-wave4/zh-cn.html, CHARLS 2018).
